# Low Infection of *Phelipanche aegyptiaca* in Micro-Tom Mutants Deficient in *CAROTENOID* *CLEAVAGE DIOXYGENASE 8*

**DOI:** 10.3390/ijms19092645

**Published:** 2018-09-06

**Authors:** Shoko Hasegawa, Takuya Tsutsumi, Shunsuke Fukushima, Yoshihiro Okabe, Junna Saito, Mina Katayama, Masato Shindo, Yusuke Yamada, Koichiro Shimomura, Kaori Yoneyama, Kohki Akiyama, Koh Aoki, Tohru Ariizumi, Hiroshi Ezura, Shinjiro Yamaguchi, Mikihisa Umehara

**Affiliations:** 1 Graduate School of Life Sciences, Toyo University, Itakura-machi, Ora-gun, Gunma 374-0193, Japan; stm4022xqi@gmail.com (S.H.); shindo.masato.03@gmail.com (M.S.); dwarf1012y.y@gmail.com (Y.Y.); shimomur@toyo.jp (K.S.); 2 Department of Applied Biosciences, Toyo University, Itakura-machi, Ora-gun, Gunma 374-0193, Japan; gq01queen@gmail.com (T.T.); fksm.ss0807@gmail.com (S.F.); 3 Faculty of Life and Environmental Sciences, University of Tsukuba, Tsukuba, Ibaraki 305-8572, Japan; yoshihiro.okabe13@gmail.com (Y.O.); ariizumi.toru.ge@u.tsukuba.ac.jp (T.A.); ezura.hiroshi.fa@u.tsukuba.ac.jp (H.E.); 4 Tsukuba Plant Innovation Research Center, University of Tsukuba, Tsukuba, Ibaraki 305-8572, Japan; 5 Graduate School of Life and Environmental Sciences, Osaka Prefecture University, Naka-ku, Sakai, Osaka 599-8531, Japan; syc02042@edu.osakafu-u.ac.jp (J.S.); akiyama@biochem.osakafu-u.ac.jp (K.Ak.); kaoki@plant.osakafu-u.ac.jp (K.Ao.); 6 College of Life, Environment, and Advanced Sciences, Osaka Prefecture University, Naka-ku, Sakai, Osaka 599-8531, Japan; ymy.mk.ymy@gmail.com; 7 Graduate School of Agriculture, Ehime University, Matsuyama, Ehime 790-8566, Japan; yoneyama.kaori.wx@ehime-u.ac.jp; 8 Precursory Research for Embryonic Science and Technology (PRESTO), Japan Science and Technology Agency, Kawaguchi, Saitama 332-0012, Japan; 9 Graduate School of Life Sciences, Tohoku University, Katahira, Aoba-ku, Sendai 980-8577, Japan; shinjiro@m.tohoku.ac.jp

**Keywords:** carotenoid cleavage dioxygenase 8, *Orobanche minor*, *Phelipanche aegyptiaca*, shoot branching, *Solanum lycopersicum*, strigolactones

## Abstract

Strigolactones (SLs), a group of plant hormones, induce germination of root-parasitic plants and inhibit shoot branching in many plants. Shoot branching is an important trait that affects the number and quality of flowers and fruits. Root-parasitic plants, such as *Phelipanche* spp., infect tomato roots and cause economic damage in Europe and North Africa—hence why resistant tomato cultivars are needed. In this study, we found *carotenoid cleavage dioxygenase 8-*defective mutants of Micro-Tom tomato (*slccd8*) by the “targeting induced local lesions in genomes” (TILLING) method. The mutants showed excess branching, which was suppressed by exogenously applied SL. Grafting shoot scions of the *slccd8* mutants onto wild-type (WT) rootstocks restored normal branching in the scions. The levels of endogenous orobanchol and solanacol in WT were enough detectable, whereas that in the *slccd8* mutants were below the detection limit of quantification analysis. Accordingly, root exudates of the *slccd8* mutants hardly stimulated seed germination of root parasitic plants. In addition, SL deficiency did not critically affect the fruit traits of Micro-Tom. Using a rhizotron system, we also found that *Phelipanche aegyptiaca* infection was lower in the *slccd8* mutants than in wild-type Micro-Tom because of the low germination. We propose that the *slccd8* mutants might be useful as new tomato lines resistant to *P. aegyptiaca*.

## 1. Introduction

Shoot branching determines aerial plant architecture, which is controlled by the formation of axillary buds in the axils of leaves and subsequent outgrowth [1]. Shoot branching is an important trait in agriculture and horticulture because it affects the flower numbers, as well as number and quality of fruits and seeds. Therefore, it is important to control number of branches in crop production. Whether axillary buds start to grow or remain dormant is governed by environmental and endogenous cues [2]. The main endogenous cues are plant hormones; cytokinin activate axillary bud outgrowth, whereas auxin, which is transported basipetally, inhibits it [3]. Strigolactones (SLs) also inhibit axillary bud outgrowth [4,5]. Several types of natural SLs have been found in root exudates of diverse plant species [6].

SLs are synthesized from β-carotene in plastids. β-Carotene is first converted to carlactone (CL) through consecutive reactions catalyzed by the β-carotene isomerase and carotenoid cleavage dioxygenases 7 and 8 (CCD7 and CCD8) [7,8]. In rice and pea, *CCD8* expression is regulated by negative feedback through SLs [9,10]. In Arabidopsis, CL is converted to a non-canonical SL, carlactonoic acid, via oxidation by CYP711A1, a cytochrome P450 encoded by *MORE AXILLARY GROWTH1* (*MAX1*) [11]. In rice, CYP711A2 catalyzes the oxidation of CL into a canonical SL, 4-deoxyorobanchol (4-DO), and CYP711A3 catalyzes the hydroxylation of 4-DO to produce the strigolactone orobanchol [12].

SL production is induced in roots under inorganic phosphate (Pi) deficiency in several plant species, including tomato, red clover, sorghum, *Lotus japonicus*, alfalfa, and rice [4,13,14,15,16]. In wild-type (WT) rice, shoot branching is not inhibited under Pi-sufficient conditions, because SL levels are low in roots [17]. Under Pi deficiency, SL levels in roots are highly elevated and SLs are probably transported to axillary buds, where they inhibit bud outgrowth [17]. Furthermore, SLs are released into soil where they likely stimulate symbiotic interactions with arbuscular mycorrhizal fungi, which supply Pi to the host plants [18]. In SL mutants of rice and Arabidopsis, shoot branching is not suppressed under Pi deficiency [17,19]. Therefore, SLs are thought to be key regulators of efficient Pi allocation and adaptation to Pi deficiency [20]. Recently, SL production is shown to be induced in response to nitrogen and sulfate deficiencies, as well as Pi deficiency in rice [21,22].

Tomato (*Solanum lycopersicum* L.), a major horticultural crop in the Solanaceae, is a model system for fruit development with a sequenced genome [23]. Tomato produces several orobanchol-type SLs, including orobanchol, solanacol, didehydro-orobanchol, orobanchyl acetate, 7-oxoorobanchol, and 7-hydroxyorobanchol [24]. Knock-down tomato lines of *CCD7* and *CCD8* have been generated by antisense introgression and RNAi-mediated silencing, respectively [25,26]. Recently, the *max1* mutant with defective CYP711 of tomato has been characterized [27]. In these low-SL lines, plant height is reduced, and the number of branches is increased. In addition, flower size, seed number, and fruit yield are reduced in *SlCCD8* knock-down lines, indicating that SLs contribute to crop yield [25].

SLs were originally characterized as germination inducers in root-parasitic plants [28,29]. SLs are released from roots into soil and induce germination of *Striga*, *Orobanche*, and *Phelipanche* species [30,31]. *Phelipanche ramosa* and *Phelipanche aegyptiaca* parasitize the roots of important crops, including tomato, and cause severe economic damage in some areas around the Mediterranean [32]. Therefore, new tomato cultivars resistant to root-parasitic plants are needed in these areas.

Previously, we demonstrated that few *Striga hermonthica* plants infected roots of the *CCD8*-defective rice mutant *d10* [4]. In this study, to evaluate the infection of SL biosynthesis–defective mutants with *P. aegyptiaca*, we found two *CCD8*-defective mutant lines, 2757 and 5291, in Micro-Tom tomato by using the “targeting-induced local lesions in genomes” (TILLING) method (Okabe et al. 2011). We characterized that both mutants had more shoot branches than WT and no detectable endogenous SLs. Exogenously applied GR24 (a synthetic SL analog used for evaluation of physiological effects of SLs; [33]) and grafting onto WT rootstock suppressed the excess outgrowth of axillary buds in the mutants. Few seeds of *P. aegyptiaca* and *Orobanche minor* germinated in root exudates of the mutants. Furthermore, *P. aegyptiaca* infection was lower in 5291 roots than in WT roots because of the low germination of *P. aegyptiaca*.

## 2. Results

### 2.1. Screening for SlCCD8-Defective Micro-Tom Mutants

Among ca. 9000 mutant Micro-Tom lines stocked in the University of Tsukuba within the framework of National Bio-Resource Project Tomato (Saito et al. 2011), we found 12 candidates for *slccd*8 mutants (Table S1) including lines 8245 and 7311 having the same point mutation in the *SlCCD8* gene. The M_3_ plants of the 12 candidates were grown, and each mutation in each line was checked by cleaved amplified polymorphic sequence (CAPS) analysis or DNA sequencing of the *SlCCD8* gene. However, we could not find homozygous mutants in lines 7343, 7024, 7311, 5639, 7720, 2481, 5550, and 3979. Such homozygous were found in lines 8245 and 8940, but the number of branches, orobanchol levels, and germination of *O. minor* did not differ from those of WT (Table S1). In the recessive homozygous mutants of 5291 and 2757, the number of branches was higher than that in WT, orobanchol was not detected in root exudates, and the root exudates did not stimulate seed germination of *O. minor* (Table S1)*.* The mutation in 5291 was identified as a change of C to T at +2578th nucleotide from the transcriptional start, resulting in a change of 494th amino acid serine to phenylalanine (Table S1). The mutation in 2757 was identified as a change of G to A at +2616th nucleotide, resulting in a change of 507th glutamic acid to lysine (Table S1). In both mutants, these mutations were located in the fifth exon of *SlCCD8* (Figure S1A). Homozygous mutants of 5291 and 2757 were propagated, checked by CAPS analysis with specific primers (Table S2, Figure S1B), and used for following experiments. When the amino acid sequences of CCD8 were compared in some plant species, 494th serine and 507th glutamic acid were conserved (Figure S2).

### 2.2. SL Levels Are Reduced in the slccd8 Mutants 5291 and 2757

**Figure 1:**
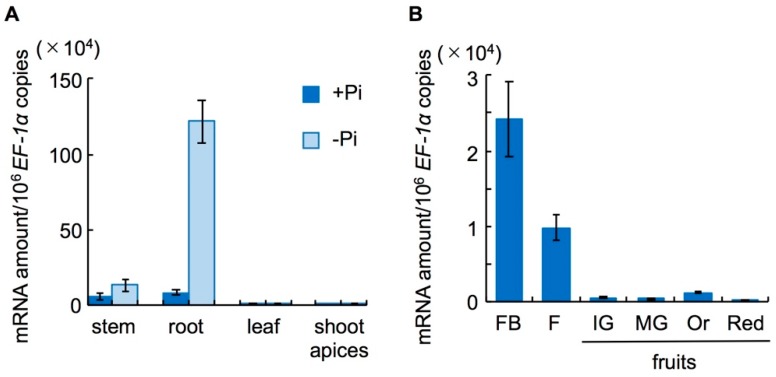
Quantitative real-time PCR (qRT-PCR) analysis of *SlCCD8* expression in wild-type (WT) Micro-Tom tomato. (**A**) Twenty-day-old seedlings. (**B**) Mature 120-day-old plants. FB, flower bud; F, flower; IG, immature green fruits; MG, mature green fruits; Or, orange fruits; Red, red fruits. Error bars, Standard error (S.E.) (*n* = 4).

To investigate in which tissues of WT Micro-Tom *SlCCD8* is strongly expressed, we performed quantitative real-time PCR (qRT-PCR) analysis using specific primers and TaqMan probes listed in Supplementary Table S2. We grew WT seedlings in the phosphate ion presence (+Pi) or absence (−Pi) of hydroponic culture medium and assayed *SlCCD8* mRNA expression in stems, roots, leaves, and shoot apices of 20-day-old seedlings (Figure 1A). *SlCCD8* expression was very low under +Pi conditions, but was elevated in stems, shoot apices, and especially in roots under −Pi conditions (Figure 1B). It was much lower in fruits than in other tissues, but slightly increased in orange fruits.

**Figure 2:**
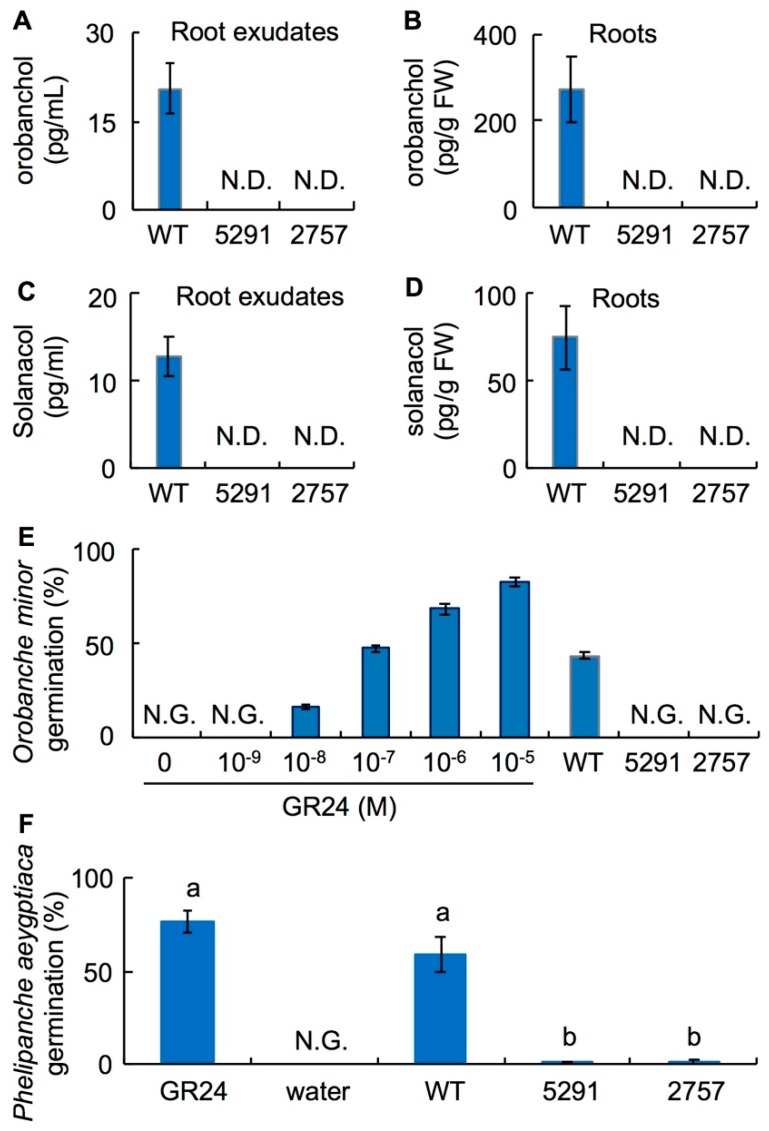
Orobanchol and solanacol levels in root exudates and roots of WT and lines 5291 and 2757, and germination assays with root-parasitic plants. (**A**) *n* = 6. (**B**) *n* = 5. (**C**) *n* = 10. (**D**) *n* = 5. (**E**,**F**) Germination assays using (**E**) *Orobanche minor* (*n* = 6) or (**F**) *Phelipanche aegyptiaca*. Error bars, S.E.; N.D., not detected; N.G., no germination.

Next, we quantified endogenous SLs in roots using liquid chromatography–tandem mass spectrometry (LC-MS/MS). Under −Pi conditions, orobanchol concentration in WT was 20.6 pg·L^−1^ in root exudates and 272.2 pg·g^−1^ FW in roots (Figure 2A,B); solanacol concentration was 12.8 pg·L^−1^ in root exudates and 74.7 pg·g^−1^ FW in roots (Figure 2C,D). In lines 5291 and 2757, these SLs were undetectable in root exudates and roots (Figure 2A–D). In germination assays, root exudates of WT seedlings induced germination of 43.4% of O. minor seeds and 59.5% of *P. aegyptiaca* seeds (Figure 2E,F). Root exudates of 5291 and 2757 did not stimulate germination of O. minor seeds (Figure 2E) and induced germination of approximately 1% of *P. aegyptiaca* seeds (Figure 2F).

### 2.3. Characterization of the Slccd8 Mutants 5291 and 2757

**Figure 3:**
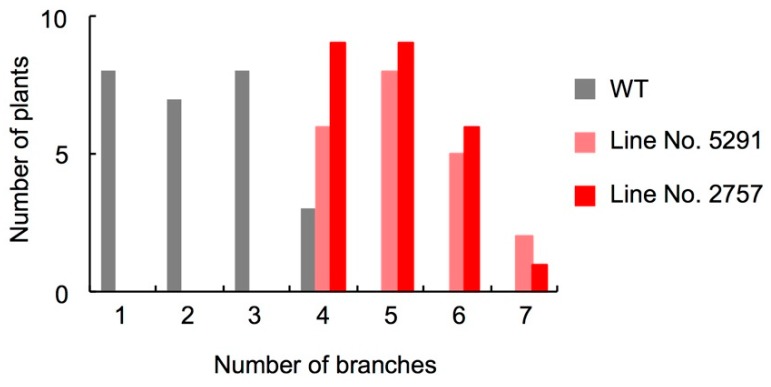
Number of branches in WT and *SlCCD8*-defective mutants. Axillary buds longer than 2 mm were counted in 30-day-old seedlings. WT, *n* = 26; line 5291, *n* = 29; line 2757, *n* = 25.

**Figure 4:**
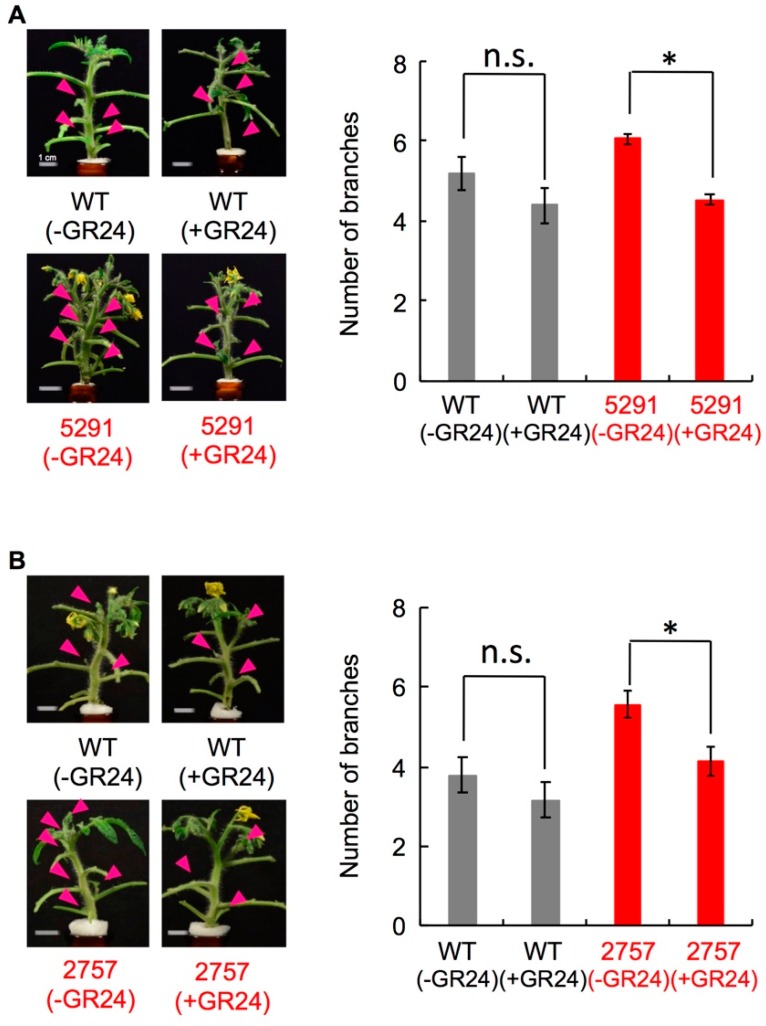
Effect of exogenously applied strigolactone (SL) on branching. GR24 (1 µM) was applied to 40-day-old seedlings of WT and (**A**) line 5291 (*n* = 3) or (**B**) line 2757 (*n* = 4). Arrowheads show outgrowing axillary buds (>2 mm). Error bars, S.E. Student’s *t*-test, * *p* < 0.05, Asterisks indicate significant differences between untreated and treated slccd8 mutants (Student’s *t*-test, * *p* < 0.05; n.s., not significant).

**Figure 5:**
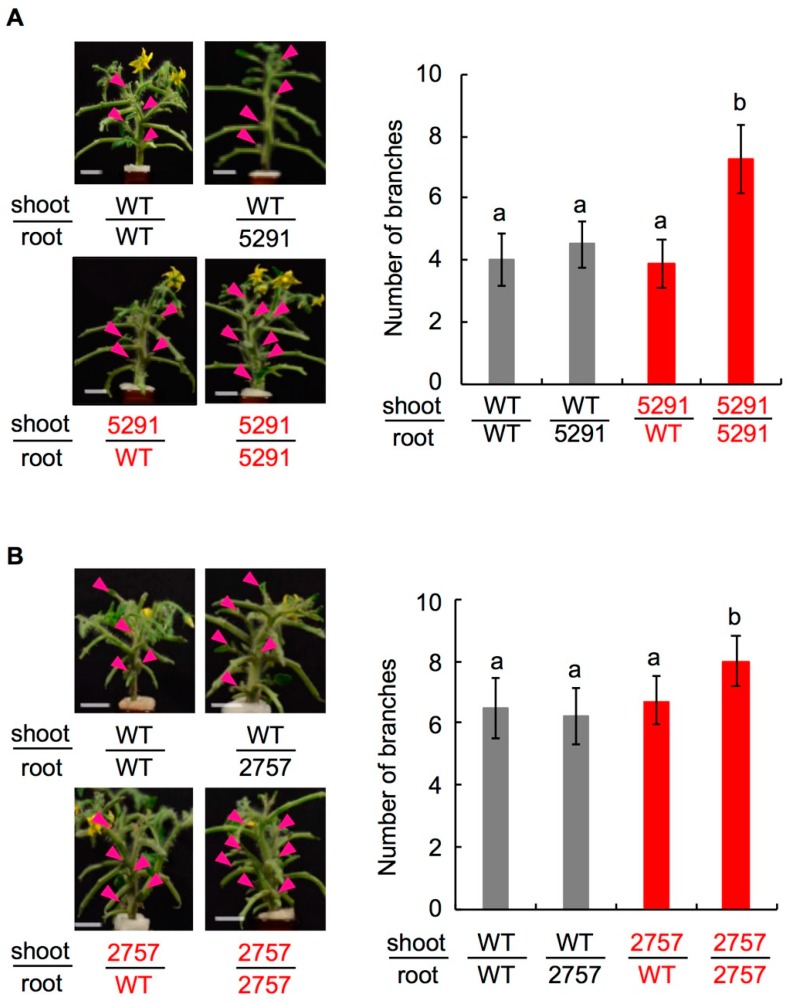
Effect of endogenous SL on branching. Photographs show 60-day-old plants grafted using scions and rootstocks from WT, and (**A**) line 5291 or (**B**) line 2757 as indicated. Arrowheads show outgrowing axillary buds. Branches were counted for 50 days. Error bars, S.D. Different lowercase letters show significant difference by Tukey’s multiple comparison test (*p* < 0.05).

**Figure 6:**
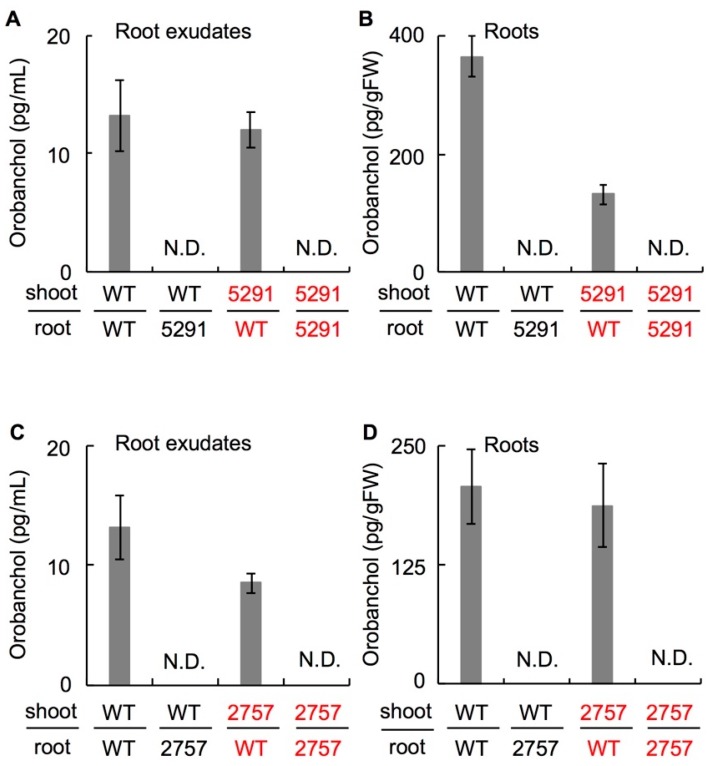
Orobanchol levels in root exudates and roots in different grafting combinations, between WT and *slccd8* mutants. (**A**) *n* = 4–9. (**B**) *n* = 4–6. (**C**) *n* = 11–18. (**D**) *n* = 6–8. Error bars, S.E.; N.D., not detected.

Among 30-day-old seedlings, WT had 1–4 axillary buds longer than 2 mm, whereas 5291 and 2757 had 4–7 (Figure 3). Exogenously applied GR24 (1 µM) suppressed axillary bud outgrowth of 5291 and 2757 to the WT levels (Figure 4, Figure S3). To evaluate the effect of endogenous SLs, we performed grafting experiments (Figure 5, Figure S4). To eliminate the effect of grafting, WT was grafted onto WT and *slccd8* mutant was grafted onto *slccd8* mutant. Grafting of WT shoot scions onto WT or *slccd8* mutant rootstocks resulted in similar numbers of branches. Grafting of *slccd8* mutant shoot scions onto WT rootstocks decreased the number of branches in comparison with that on *slccd8* mutant rootstocks. Under −Pi conditions, orobanchol and solanacol were produced in WT rootstocks, but were undetectable in *slccd8* mutant rootstocks (Figure 6, Figure S5).

In rice and Arabidopsis, *CCD8*-defective mutants have delayed leaf senescence [34,35], but no delay in leaf senescence was observed in 5291 (Figure S6). Among a number of flower and fruit traits examined (Figure S7), the number of branches increased in *slccd8* mutants (Figure 3), but the number of flowers and fruits did not increase in 2757 and it decreased in 5291; interestingly, Brix slightly (but significantly) increased in both *slccd8* mutants.

### 2.4. Phelipanche aegyptiaca Infection Is Decreased in Roots of Slccd8 Mutants

**Figure 7:**
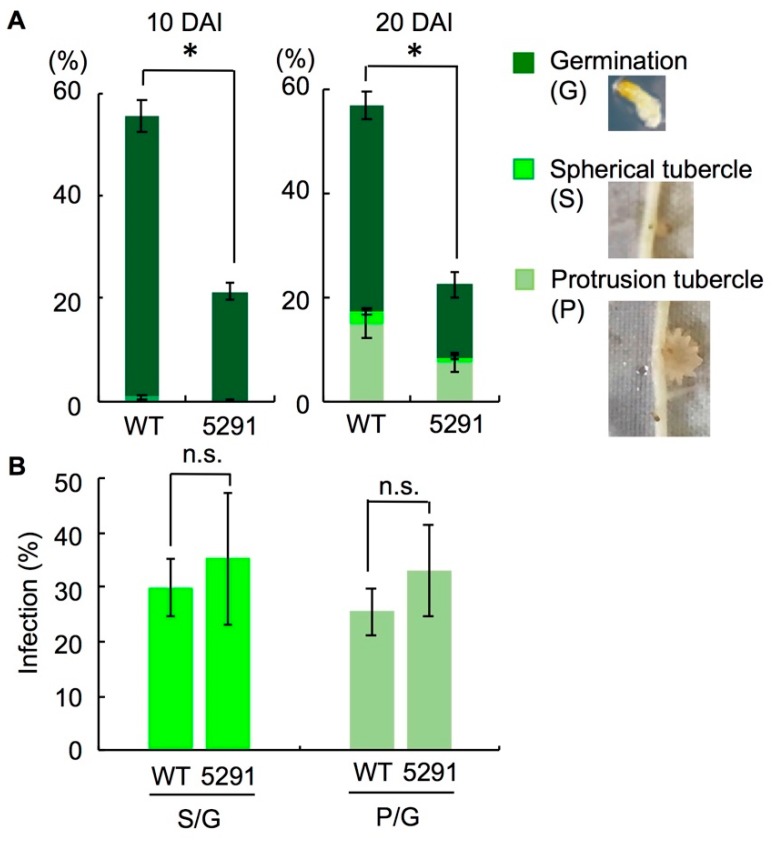
Infection assay with *Phelipanche aegyptiaca*. (**A**) Proportion of germinated *P. aegyptiaca* seeds, spherical tubercles, and protrusion tubercles 10 and 20 days after inoculation (DAI) of tomato roots (*n* = 3). (**B**) Infection with germinated *P. aegyptiaca* seeds 20 DAI (*n* = 3). S/G and P/G mean frequency of spherical tubercles and protrusion tubercles to germinated Phelipanche, respectively. Error bars, S.E.; * *p* < 0.05 (Student’s *t*-test); n.s., not significant.

To evaluate the impact of low SL levels on the interaction of Micro-Tom with root-parasitic plants, we performed an infection assay with *P. aegyptiaca*. Germination of *P. aegyptiaca* seeds was approximately 55% at 10 days after inoculation (DAI) and 57% at 20 DAI around WT roots, but was significantly lower (21% at 10 DAI and 23% at 20 DAI) around 5291 roots (Figure 7A). Some germinated *P. aegyptiaca* infected tomato roots at 20 DAI. The proportions of spherical and protrusion tubercles were 2.5% and 14.7% in WT, respectively, and 1.0% and 7.4% in 5291 (Figure 7A). There was no significant difference in the proportion of tubercle formation in *P. aegyptiaca* after germination between WT and 5291 (Figure 7B).

## 3. Discussion

CCD8 converts 9-*cis*-β-apo-10′-carotenal to carlactone in plastids [7]. This reaction is an important step to form the basic chemical structure of SLs in SL biosynthesis. The role of the *CCD8* gene has been characterized in several plant species [25,36,37,38]. Using the TILLING method, we found two *slccd8* mutants (5291 and 2757) of Micro-Tom tomato and characterized their growth traits. In both mutants, point mutations are located in the fifth exon, which is important for catalysis (Supplementary Figure S1). In WT seedlings, the highest expression of the *SlCCD*8 gene was found in roots under Pi deficiency (Figure 1). Because SL levels are elevated under Pi deficiency in tomato [15], we measured the SL levels in root exudates and roots of WT and *slccd8* mutants grown under Pi deficiency. In the *slccd8* mutants, the tomato endogenous SLs orobanchol and solanacol were undetectable (Figure 2A–D), and root exudates of the *slccd8* mutants barely induced germination of the root-parasitic plants *O. minor* and *P. aegyptiaca* (Figure 2E,F). These results indicate that SLs are barely produced in the mutants.

The number of branches was higher in the *slccd8* mutants than in WT (Figure 3) and was reduced by GR24 treatment (Figure 4, Figure S3). In the *slccd8*, axillary bud formation was not stimulated, but growth of dormant buds was activated in comparison with WT. However, this difference was smaller than that of between the knock-down lines of SL-biosynthetic genes and their parent cultivars [25,26] because plant size of Micro-Tom is small and the total number of axillary buds is low. In addition, there is no significant effect on GR24 treatment in WT of Micro-Tom because of the small size and small number of axillary buds. *SlCCD8* expression was very low in flowers and fruits (Figure 1B); no significant differences were detected in several fruit traits that we checked (Figure S7). When *slccd8* mutant shoot scions were grafted onto WT rootstocks, the number of branches in the *slccd8* mutants decreased and became similar to that of WT (Figure 5, Figure S4). This result indicates that SLs produced in roots were transported to the scions and is consistent with grafting experiments in the *rms1* mutant of pea and the *max4* mutant of Arabidopsis, which are *CCD8*-defective mutants [39,40]. SL transportation from roots to shoots is regulated by *PDR1*, which encodes an ATP-binding cassette transporter [41]. Because only small amounts of SLs were produced in WT shoot scions, SLs were hardly transported to *slccd8* mutant rootstocks (Figure 6, Figure S5).

*Phelipanche aegyptiaca*, a holoparasitic plant that infects important dicotyledonous crops, including tomato [42], is the main limiting factor in processing-tomato production in Israel and some countries of the Arabian Peninsula [43]. The annual economic losses caused by *P. aegyptiaca* infection increase continuously; infection is not completely suppressed by available herbicides [43]. A tomato mutant with decreased SL production, *Sl-ORT1*, induced by fast-neutron irradiation, is resistant to *Orobanche* and *Phelipanche* spp. [44,45]. In *Sl-ORT1*, the transcription level of *CCD7* is lower than that in WT [46], but the mutation and the mechanism of SL deficiency are unknown. The rice *CCD8*-defective mutant *d10* is infected by fewer *S. hermonthica* plants than WT [4]. Infection of *SlCCD8* RNAi lines by *P. ramosa* is 10% of that in WT seedlings [25]. Probably, small amounts of SLs were produced in roots of the RNAi lines and causes the infection of *P. ramosa*. However, these lines are more prone to infection by pre-germinated *P. ramosa* and the parasite develops faster than in WT, suggesting a positive role of SLs in host defense against parasitic plant infection [47]. The reduced levels of defense-related hormones such as jasmonic, salicylic, and abscisic acids in the *SlCCD8* RNAi lines [48] may contribute to their increased susceptibility to parasite infection.

In this study, *P. aegyptiaca* infection of the roots was lower in the *slccd8* mutants than in WT because of a significantly lower germination of *P. aegyptiaca* around the roots of the mutants (Figure 7A). In germination assay, root exudates of the *slccd8* mutants which were extracted with ethyl acetate barely stimulated germination of *P. aegyptiaca* (~1%; Figure 2F), whereas germination of *P. aegyptiaca* was approximately 23% in infection assay (Figure 7A). SLs are major germination stimulants for the roots of parasitic plants, but some *Orobanche* and *Phelipanche* spp. use other germination signals. *Orobanche cumana*, a parasite which infects sunflowers, recognizes dehydrocostus lactone [49]. *Phelipanche ramosa* responds to glucosinolate-breakdown products derived from rapeseed oil [50]. These data indicate that not only SLs, but also compounds other than SLs might be produced in Micro-Tom roots and stimulate germination of *P. aegyptiaca*. The *slccd8* mutants failed to prevent infection once *P. aegyptiaca* had germinated (Figure 7B). To completely block *P. aegyptiaca* infection in tomato, aqueous compounds inducing germination should be identified and their biosynthesis should be suppressed.

## 4. Materials and Methods

### 4.1. Plant Material and Growth Conditions

Seeds of the wild type and the *slccd8* mutant M_3_ line of Micro-Tom (*Solanum lycopersicum* L.) were provided by the University of Tsukuba. The seeds were surface-sterilized in 1% sodium hypochlorite solution containing 0.05% Tween 20 for 2 min and rinsed with sterile water five times. The sterile seeds were placed on half-strength Murashige and Skoog (MS) medium [51] containing 0.6% agar in PLANTAN culture pots (Sibata, Saitama, Japan), and cultured in a plant incubator FLI-2000 (Eyela, Tokyo, Japan) with a 16-h light/8-h dark photoperiod (63 µmol m^−2^·s^−1^) at 23 °C for 10 days. The plants were then transferred to half-strength Hoagland’s culture medium [52] with (+Pi) or without Pi (−Pi). In the grafting experiment, 10-day-old seedlings were divided into shoot scions and rootstocks. Combination of the grafting was WT scions onto WT root stocks, WT scions onto root stocks of *slccd8* mutant, *slccd8* mutant scions onto WT root stocks, and *slccd8* mutant scions onto root stocks of *slccd8* mutant. The graft junction was fixed with a silicone tube. Branches >2 mm long were counted 50 days after grafting. For evaluation of flower and fruit traits, seedlings were grown in mixed soil (vermiculite:Metro-Mix = 1:1). Brix and acidity were measured using a PAL-BX/ACID3 master kit (Atago, Tokyo, Japan).

### 4.2. Screening for slccd8 Mutants

*slccd8* mutants were screened by the TILLING method as described [53] from about 9000 mutagenized M_2_ lines [54]. Genomic DNA was extracted from M_3_ leaves (ca. 20 mg) of Micro-Tom in 400 µL of DNA extraction buffer (200 mM Tris·HCL, 250 mM NaCl, 25 mM EDTA, and 0.5% SDS, pH 7.5) using a BioMasher II homogenizer (Nippi, Inc., Tokyo, Japan). The extract was centrifuged at 8000× *g* for 1 min, the supernatant (300 µL) was transferred to a new 1.5-mL tube, and 2-propanol (300 µL) was added and gently mixed for 2 min. The mixture was centrifuged at 11,000 rpm for 5 min, the supernatant was removed, and the pellet was dissolved in 100 µL of TE. from M_2_ leaves, and PCR was performed using 5′ end-IRDye-labeled *slccd8*-specific primers to amplify the desired region. PCR primers for TILLING were designed in Genetyx v. 10 software (Genetyx corporation, Tokyo, Japan) and are shown in Supplementary Table S2. PCR conditions were as follows: Initial denaturation at 95 °C for 2 min; 40 cycles of 95 °C for 30 s, 60 °C for 30 s, and 72 °C for 1 min; and a final extension at 72 °C for 5 min. Heteroduplexes of the PCR products were formed by heating at 95 °C for 7 min and gradual cooling to 4 °C. Recombinant SlENDO1 nuclease was used to cleave the base mismatches at 45 °C for 20 min [55]. Digested fragments were purified using Sephadex-G50 gel and concentrated at 85 °C for 80 min. TILLING screening was performed using a LI-COR DNA analyzer (LI-COR Biotechnology, Lincoln, NE, USA) as described [56].

### 4.3. CAPS Method

Genomic DNA was extracted from young leaves (ca. 20 mg) of Micro-Tom as above. PCR was carried out with 5291- and 2757-specific primers (Supplementary Table S2). For screening of recessive homozygous mutants, the amplified PCR products were digested with *Hpy*188I and *Mbo*II, respectively. The difference in restriction fragment patterns between WT and mutants was confirmed by 2% agarose electrophoresis.

### 4.4. Analysis SlCCD8 Gene Expression

Total RNA was extracted from stems, roots, leaves, shoot apexes, flower buds, flowers, and fruits, using an RNeasy Plant Mini Kit (Qiagen, Hilden, Germany). RNA concentration was measured using a NanoDrop 200c spectrophotometer (Agilent Technologies, Santa Clara, CA, USA). An aliquot (200 ng) was used for cDNA synthesis with a ReverTra Ace qPCR RT Kit (Toyobo, Osaka, Japan). qRT-PCR was performed using a Thunderbird Probe qPCR Mix (Toyobo), specific primers, and probes listed in Supplementary Table S2. Expression of SL-related genes was analyzed by a real time PCR system StepOnePlus (Thermo Fisher Scientific, Waltham, MA, USA). The SlEF1-α gene was used as an internal standard [57,58].

### 4.5. Chemicals

rac-GR24 was purchased from Chiralix (Nijmegen, The Netherlands). Orobanchol and solanacol were synthesized as described [59,60]. [6′-D_1_]-Orobanchol was described in [61]. [6′-^13^C_1_]-solanacol ((±)-(3aR*,4R*,8bR*)-4-Hydroxy-7,8-dimethyl-3,3a,4,8b-tetrahydro-2H-indeno[1,2-b]furan-2-one) was synthesized as reported (Figure S8) [59]. Ester condensation of the racemic 4-hydroxy ABC lactone with methyl [1-^13^C] formate by the use of sodium hydride in *N*,*N*-dimethylformamide followed by alkylation with racemic 4-bromo-2-methyl-2-buten-4-olide provided (±)-[6′-^13^C_1_]-solanacol and (±)-[6′-^13^C_1_]-2′-epi-solanacol. (±)-[6′-^13^C_1_]-Solanacol was purified by a silica gel column (Kieselgel 60, Merck, n-hexane-ethyl acetate stepwise) and semi-preparative HPLC (Inertsil SIL-100A, GL Sciences, 20% ethanol in n-hexane). NMR spectra were obtained with a JEOL JNM-ECZ500R NMR spectrometer. Chemical shifts were referenced to tetramethylsilane as an internal standard. Mass spectra were recorded on an Waters Xevo QTof MS mass spectrometer. **(±)-[6′-^13^C_1_]-Solanacol**: ^1^H-NMR (CDCl_3_, 500 MHz) δ: 2.05 (3H, br s, H-7′), 2.30 (3H, s, H-9), 2.36 (3H, s, H-10), 3.82 (1H, m, H-3a), 5.25 (1H, br s, H-4), 6.16 (1H, d, J = 7.5 Hz, H-8b), 6.23 (1H, m, H-2′), 7.00 (1H, m, H-3′), 7.17 (1H, d, J = 7.7 Hz, H-6), 7.24 (1H, d, J = 7.7 Hz, H-5), 7.56 (1H, dd, ^1^J_CH_ = 186.2 Hz, ^4^J_HH_ = 2.3 Hz, H-6′); HR-ESI-TOF-MS *m*/*z*: 344.1234 [M + H]^+^ (calcd. for C_18_^13^CH_19_O_6_, *m*/*z*: 344.1215).

### 4.6. Quantitative Analysis of SLs by LC-MS/MS

Hydroponic culture medium (10 mL) was collected, [6′-D_1_]-orobanchol and [6′-^13^C]-solanacol (200 pg each) were added as internal standards and the medium was extracted with 4 mL of ethyl acetate twice. The ethyl acetate phase was evaporated to dryness under nitrogen and dissolved in ethyl acetate: n-hexane (35:65). The extracts were loaded onto Sep-Pak Silica 1-mL cartridges (Waters, Milford, MA, USA) and washed with ethyl acetate: n-hexane (35:65), and SLs were then eluted with ethyl acetate: n-hexane (50:50). To measure endogenous SL levels in roots, the roots (0.1–0.5 g) were homogenized in 5 mL of acetone containing [6′-D_1_]-orobanchol and [6′-^13^C]-solanacol (200 pg each) and filtered to remove the residues. The filtrates were evaporated to dryness under nitrogen, dissolved in 4 mL of Milli-Q water, added 20 µL of 1 N HCl, and extracted with 4 mL of ethyl acetate twice. The ethyl acetate phase was evaporated to dryness under nitrogen. The extracts were dissolved in 10% acetone and loaded onto Oasis HLB 3-mL cartridges (Waters) and eluted with 3 mL of 60% acetone twice. The eluates were evaporated to dryness under nitrogen, dissolved in ethyl acetate: n-hexane (35:65), and chromatographed on Sep-Pak Silica 1-mL cartridges as above. SL fractions were dissolved in 50% acetonitrile and analyzed by LC-MS/MS on a quadrupole tandem mass spectrometer (3200 QTRAP; Sciex, Framingham, MA, USA) and a high-performance liquid chromatograph (Prominence; Shimadzu, Kyoto, Japan) equipped with a reverse-phase column (Acquity UPLC BEH-C18, ø2.1 mm × 50 mm, 1.7 µm; Waters). Qualitative analysis of the data was performed in Analyst v. 1.5.1 software, and fragment analysis was performed in MultiQuant v. 2.0.2 software (both from Sciex).

### 4.7. Germination of Root-Parasitic Plant Seeds

Germination assays with *O. minor* and *P. aegyptiaca* seeds were performed as described [16]. Culture medium (10 mL) was collected after 2 weeks of hydroponic culture without Pi and extracted with 4 mL of ethyl acetate twice. The ethyl acetate layer was evaporated to dryness under nitrogen. The samples were then dissolved in 600 µL of Milli-Q water. Seeds of parasites were surface sterilized in 1% sodium hypochlorite containing 0.05% Tween 20 for 2 min and conditioned at 23 °C for 7 days on moist glass fiber filters (GF/A, Whatman, Maidstone, UK). The filters were moved to each well of a 96-well plate after conditioning, 20 µL of 1 × 10^−5^–1 × 10^−9^ M GR24 solution, Milli-Q water, or the sample solution was added to each well, and the seeds were incubated at 23 °C for 7 days. Germinated seeds were counted microscopically.

### 4.8. Measurement of Membrane Ion Leakage and Chlorophyll Contents

Micro-Tom leaf disks were collected 24 days after sowing and floated on 2.5 mM MES buffer (pH 5.7) containing 0.05% Tween 20 for 0–16 days. After incubation, the buffer was diluted to one-third of its initial concentration with Milli-Q water, and electrical conductivity was measured using an ES-51 electrical conductivity meter (Horiba, Kyoto, Japan). The leaf segments were placed in 1.5-mL tubes containing zirconia beads, frozen in liquid nitrogen, and crushed in a vortex mixer. Chlorophylls were extracted from each leaf segment in 80% acetone. Absorbance at 663.2 and 646.8 nm was measured in a DU720 spectrometer (Beckman Coulter, La Brea, CA, USA). Chlorophyll contents were calculated as described [62].

### 4.9. Root Infection with Phelipanche aegyptiaca

Infection assay with *P. aegyptiaca* was performed according to [63]. The seeds were surface sterilized, allowed to imbibe on filter paper for 7 days at 25 °C, and then cultured on MS medium containing 3% sucrose and 0.8% agar for 4 days. WT and line 5291 seeds were allowed to imbibe on filter paper for 5 days and were then cultured Hoagland’s culture medium for 4 weeks. Twenty *P. aegyptiaca* seeds were placed around one host root. Germination rate was evaluated by using 200 seeds each with three replicates. We calculated the proportion of germination and the formation of spherical and protrusion-like tubercles at 10 and 20 DAI.

## Supplementary Materials

Supplementary materials can be found at http://www.mdpi.com/1422-0067/19/9/2645/s1.

## Abbreviations

4DO	4-deoxyorobanchol
CAPS	cleaved amplified polymorphic sequence
CCD	carotenoid cleavage dioxygenase
CL	carlactone
DAI	day after innoculation
LC-MS/MS	liquid chromatography-tandem mass spectrometry
MS	Murashige and Skoog
N.D.	not detected
N.G.	no germination
n.s.	not significant
Pi	inorganic phosphate
S.E.	standard error
SL	strigolactone
TILLING	targeting-induced local lesions in genome
WT	wild-type
