# Supplementary material for: Low Infection of Phelipanche aegyptiaca in Micro-Tom Mutants Deficient in CAROTENOID CLEAVAGE DIOXYGENASE 8
Source: Int J Mol Sci. 2018 Sep 6;19(9):2645. doi: 10.3390/ijms19092645 (PMC6163878; doi:10.3390/ijms19092645)
Supplement: Supplementary file 1 [file ijms-19-02645-s001.zip › ijms-347930-SI.pdf]

Supplementary Table S1 *SlCCD8* mutant lines provided by the University of Tsukuba

Line No.	Point mutation (nucleotide)	Point mutation (amino acid)	Recessive homozygous mutants	Number of branches	Orobanchol	Germination assay
WT	–	–	–	1–4	Detected	Germinated
7343	G1498T	V206F	Not found	–	–	–
7024	A1507T	T209S	Not found	–	–	–
8245	G1624A	D248N	Found	1–4	Detected	Germinated
7311	G1624A	D248N	Not found	–	–	–
5639	C1769T	P296L	Not found	–	–	–
7720	C2122T	D387 = Stop codon	Not found	–	–	–
8940	G2214T	Non-coding	Found	1–4	Detected	Germinated
2481	G2266A	Splice junction	Not found	–	–	–
5550	G2271A	G414R	Not found	–	–	–
3979	C2302T	P424L	Not found	–	–	–
5291	C2578T	S494F	Found	4–7	Not detected	Not germinated
2757	G2616A	E507K	Found	4–7	Not detected	Not germinated

Supplementary Table S2 Primers used in this study

Primer name*	5′-sequence-3′
SlCCD8-TILLING F	AACCTCATTCCACATCATGTCAC
SlCCD8-TILLING R	TTGGAACCCAACAACCATGTAG
5291 F	AAGGCATGGATATGTGCAGT
5291 R	TTGTTGAGAACAGGGCCAAA
2757 F	AAGGTCAGCTTCTTTTCCTTAC
2757 R	TGTTACACCTTGTTCAGAACAG
SlCCD8-qRT-PCR F	AGATATGCTTATGCTTGTGGTGCTA
SlCCD8-qRT-PCR R	ACAGCACCTTCATCATACCAATTCT
SlCCD8-qRT-PCR TaqMan	f-GTCCCCAACACCCTCACCAAGATTGATTTAT-t**
SlEF1α-qRT-PCR F	CCAAGAGGCCATCAGACAAA
SlEF1α-qRT-PCR R	AGGCTTGATCACACCAGTCTCA
SlEF1α-qRT-PCR TaqMan	f-GCCCTCCGTCTTCCACTTCAG-t**

*F and R indicate forward and reverse primers, respectively.

**f and t in TaqMan probe sequences indicate the fluorescence labels FAM and TAMRA, respectively.

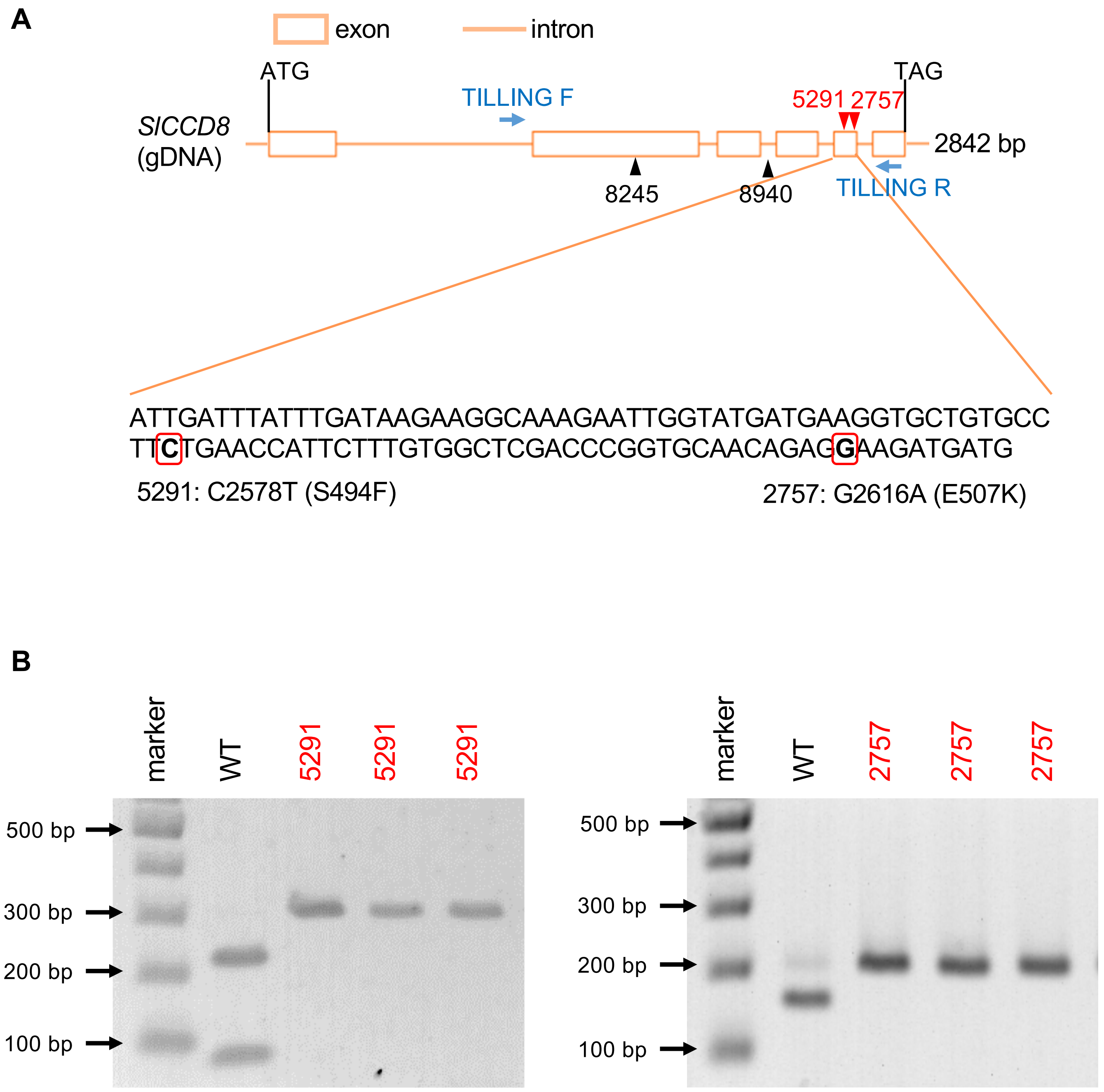

Supplementary Fig. S1 Detection of lines 5291 and 2757. **A**. Structure of the *SlCCD8* gene. **B**. The *slccd8* mutants were found using the CAPS method. *Hpy*188I was used for 5291 and *Mbo*II for 2757. Red numbers indicate *slccd8* recessive homozygous mutants.

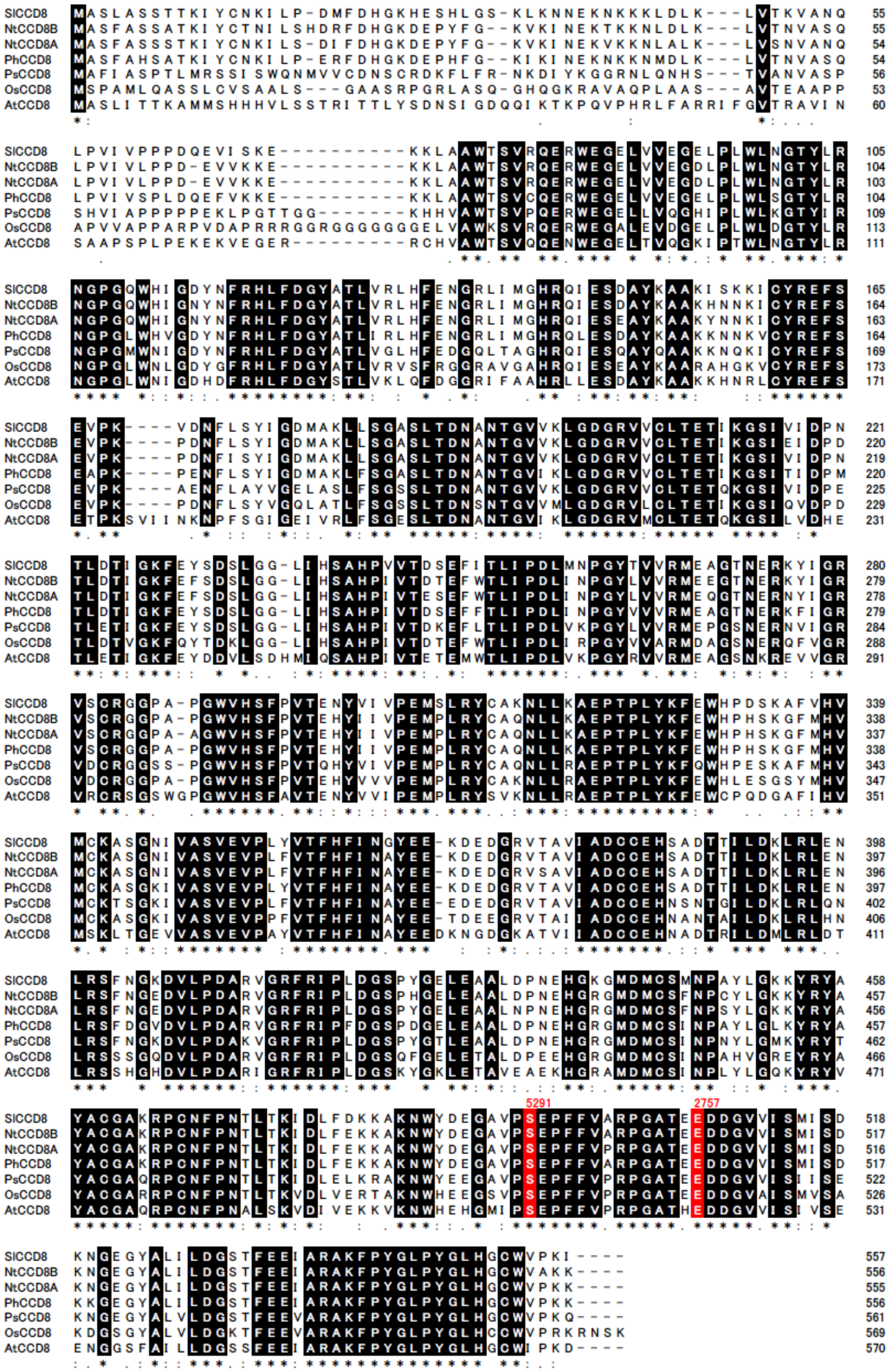

Supplementary Fig. S2 Alignment of representative CCD8 amino acid sequences. Sl, *Solanum lycopersicum*; Nt, *Nicotiana tabacum*; Ph, *Petunia hybrida*; Ps, *Pisum sativam*; Os, *Oryza sativa*; At, *Arabidopsis thaliana*.

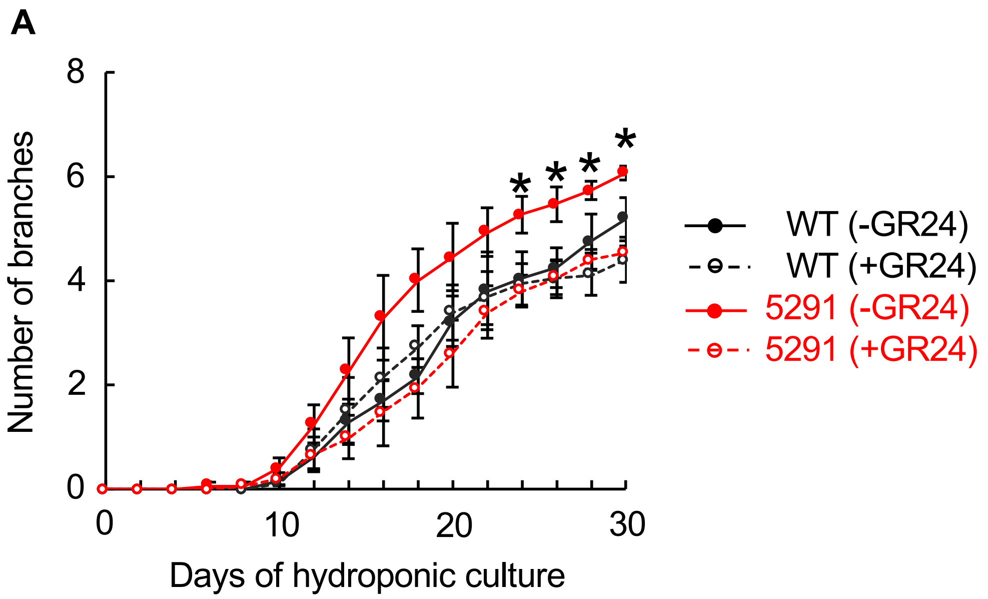

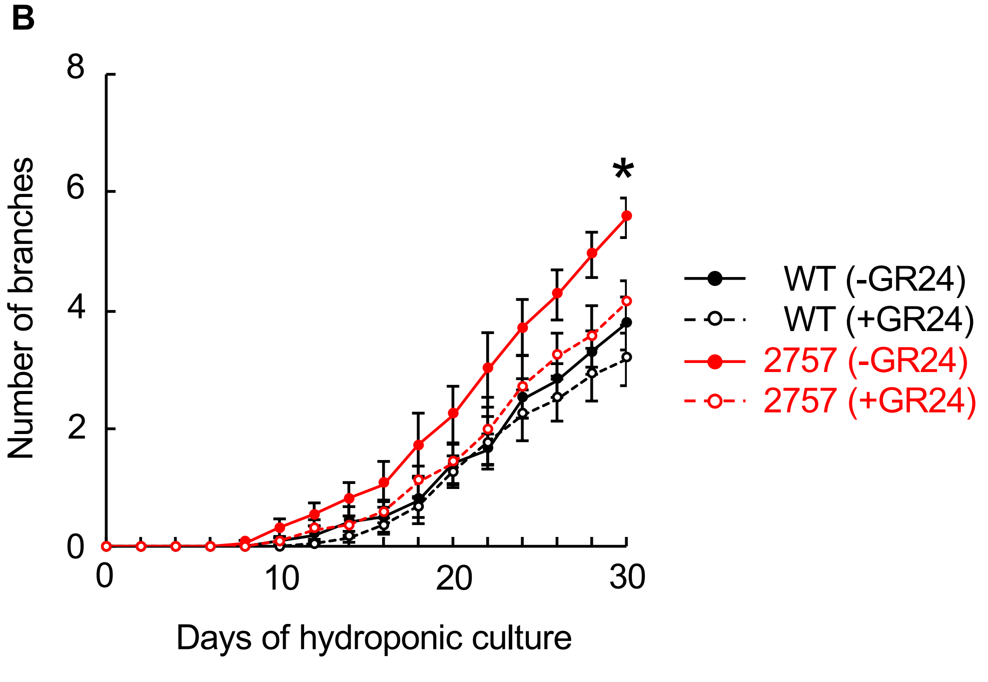

Supplementary Fig. S3 Effect of exogenously applied SL (GR24) on branching. Branches were counted in 40-day-old plants. **A**. WT, *n* = 3; line 5291, *n* = 3. **B**. WT, *n* = 4; line 2757, *n* = 4. Error bars, S.E. Asterisks indicate significant differences between untreated and treated *slccd8* mutants (Student's *t*-test, *$P < 0.05$).

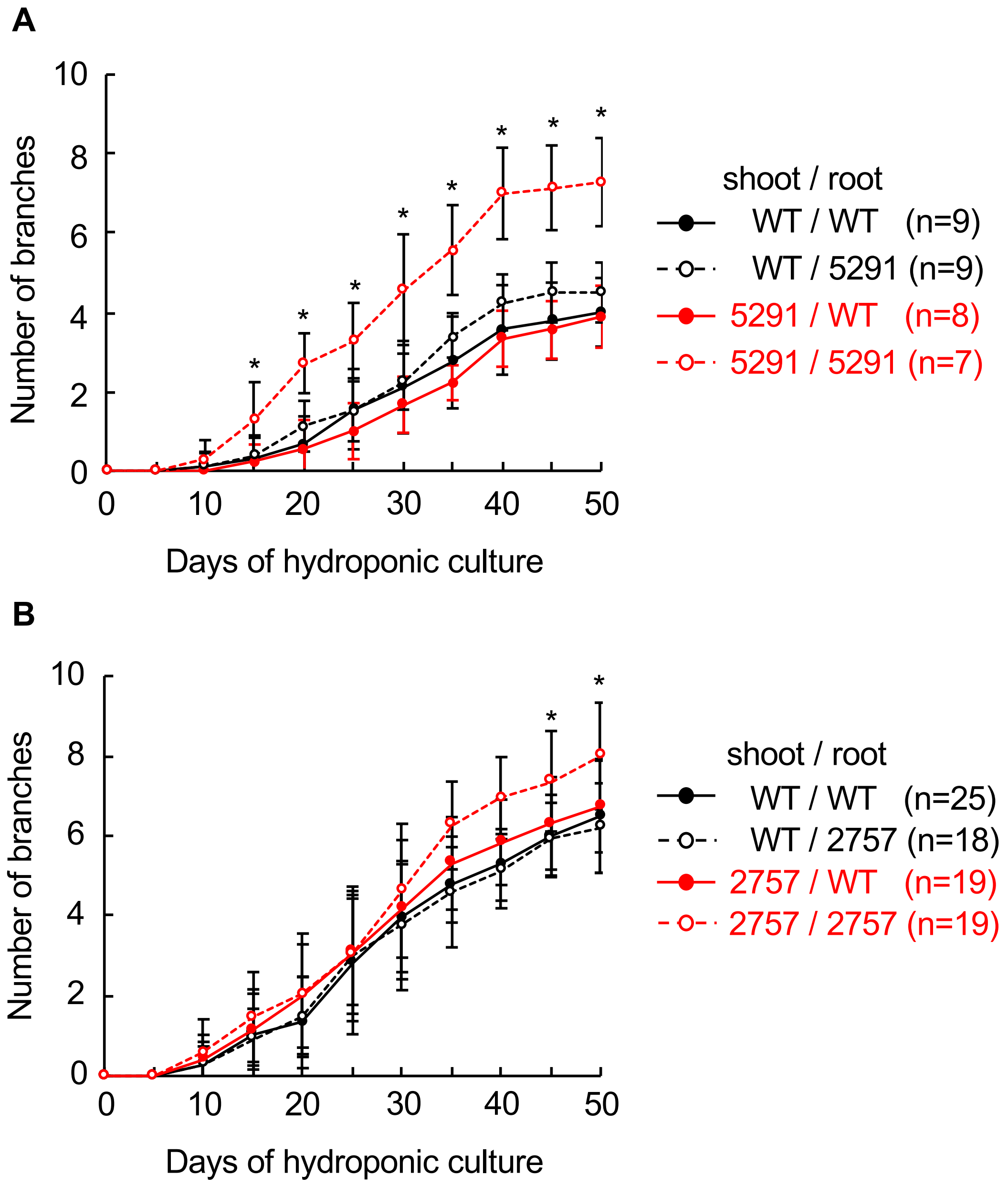

Supplementary Fig. S4 Effect of endogenous SL on branching. Branches were counted for 50 days. **A**. Grafting of WT and line 5291. **B**. Grafting of WT and line 2757. Error bars, S.D. Asterisks indicate significant differences between 5291/5291 and 5291/WT in B and between 2757/2757 and 2757/WT in D (Student's *t*-test, *$P < 0.05$).

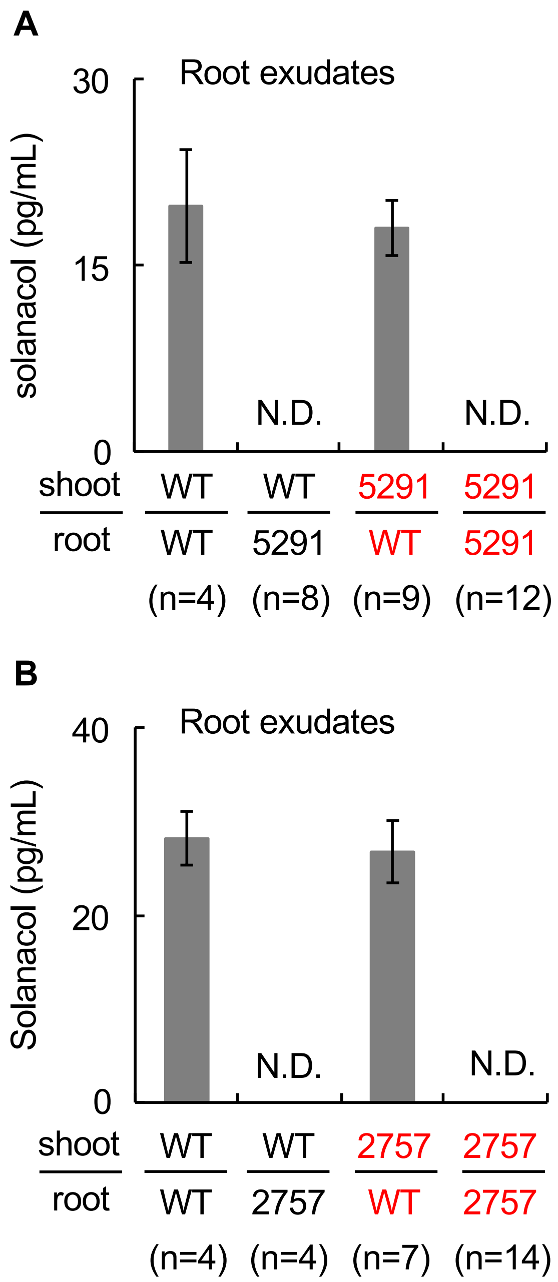

Supplementary Fig. S5 Solanacol levels in root exudates in different grafting combinations between WT and *slccd8* mutants. **A** Grafting of WT and line 5291. **B**. Grafting of WT and line 2757. Error bars, S.E.; N.D., not detected.

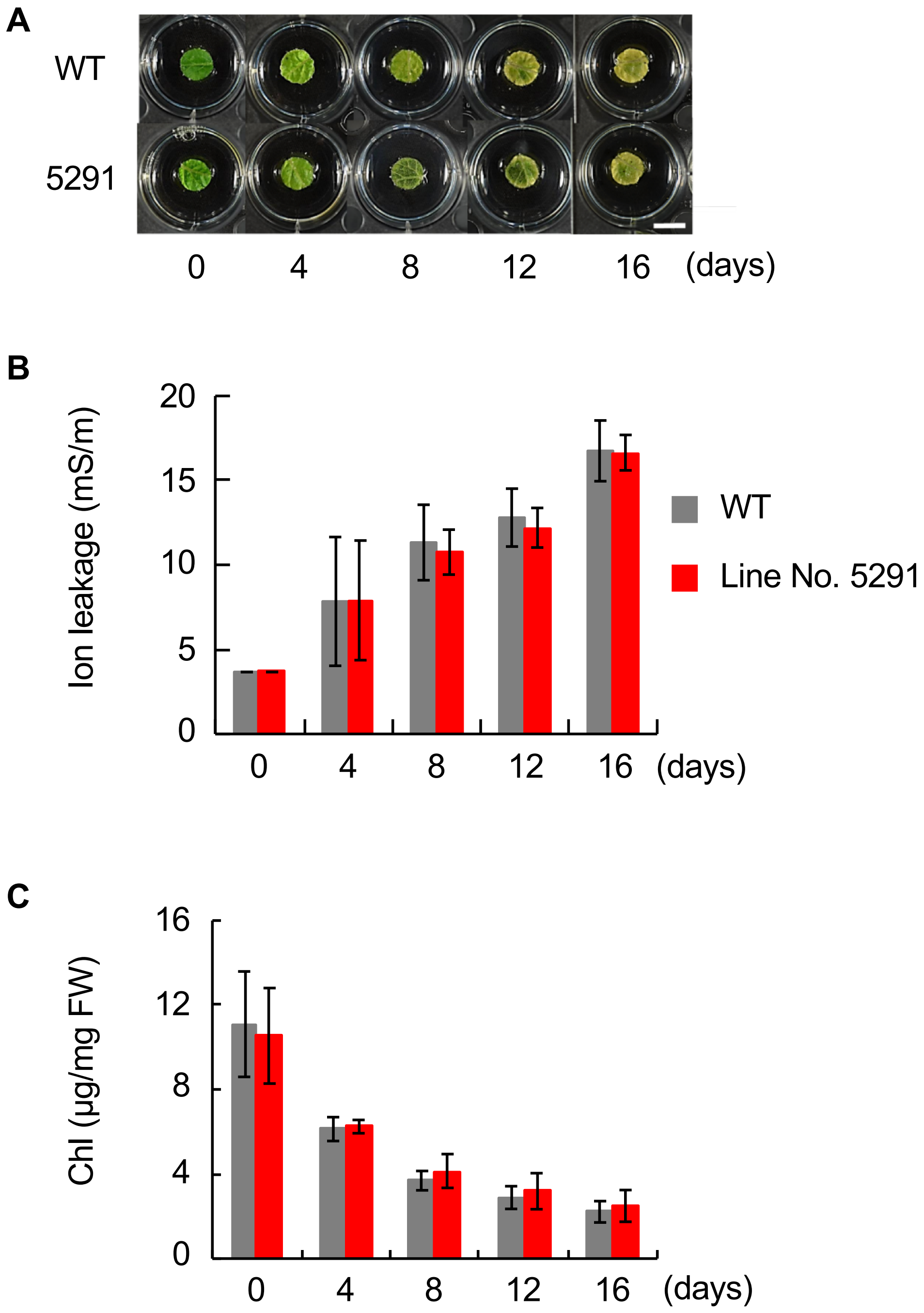

Supplementary Fig. S6 Effect of exogenously applied SL on leaf senescence in WT and line 5291. **A**. Leaf discs. **B**. Ion leakage. **C**. Chlorophyll content. Error bars, S.E. (n = 3). Student's *t*-test, No significant differences were found in Student's *t*-test ($P \geq 0.05$).

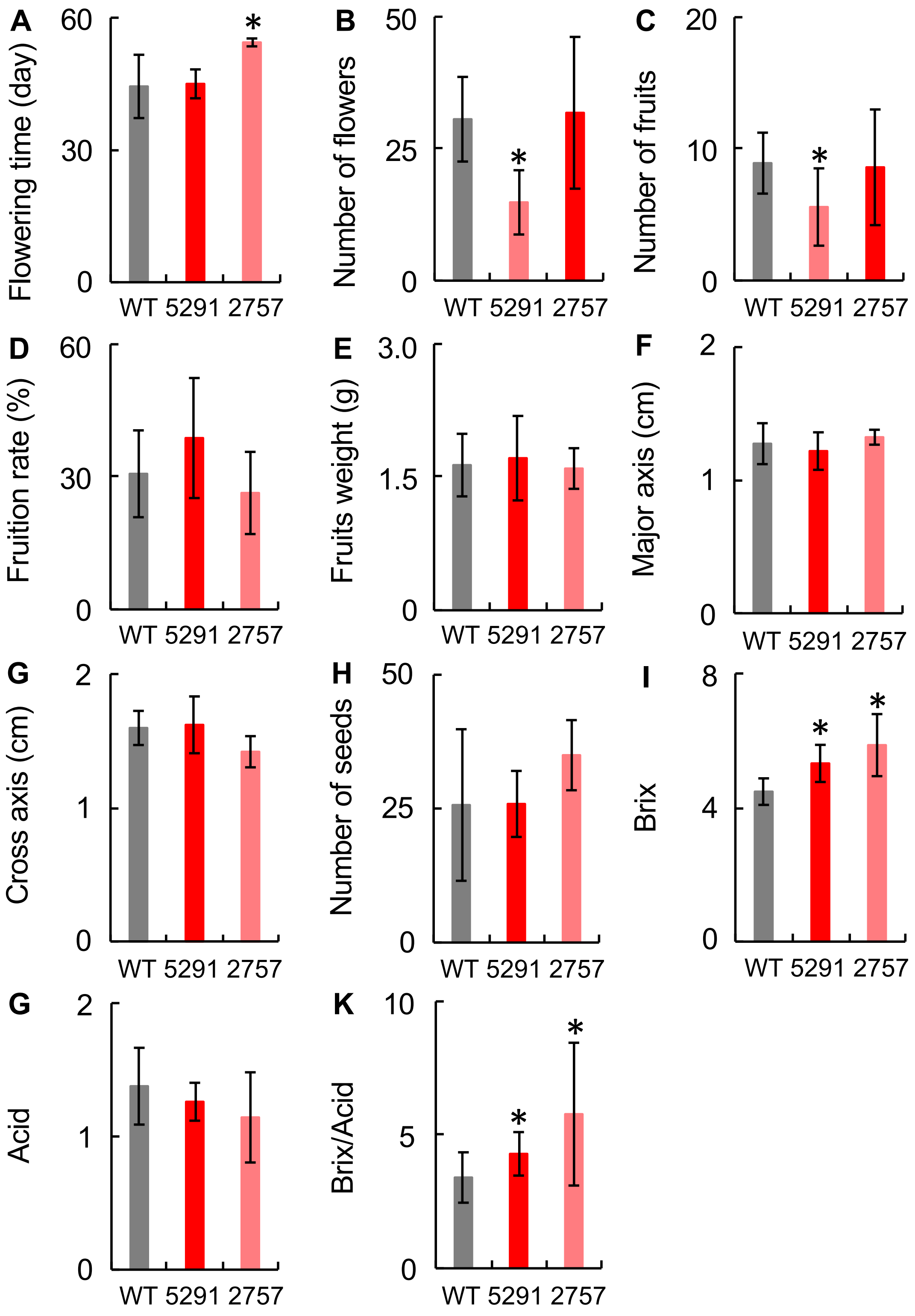

Supplementary Fig. S7 Flower and fruit traits in *slccd8* mutants. Fruition rate is the number of fruits divided by the number of flowers. All fruit traits were assessed 70 days after flowering. WT and 5291, n = 12; 2757, n = 5. Error bars, S.D. Student's t-test, $*P < 0.05$.

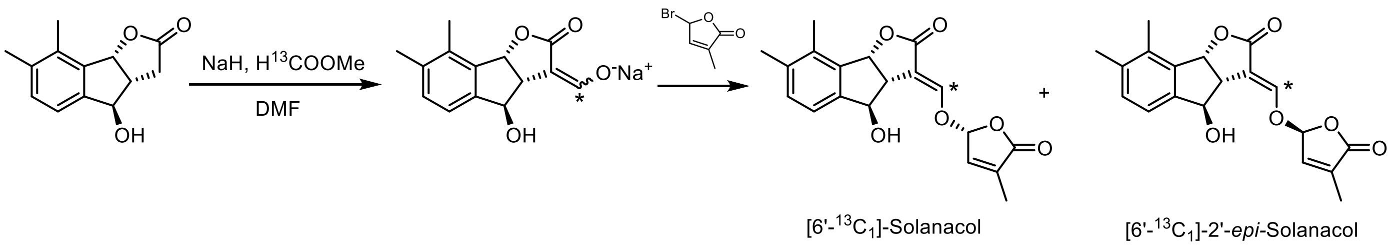

Supplementary Fig. S8 Synthesis of [6'-$^{13}C_1$]-solanacol. Asterisks indicate the position of ^{13}C.
